# Lung Stereotactic Body Radiation Therapy (SBRT) dose gradient and PTV volume: a retrospective multi-center analysis

**DOI:** 10.1186/s13014-019-1334-9

**Published:** 2019-09-03

**Authors:** David Hoffman, Irena Dragojević, Jeremy Hoisak, David Hoopes, Ryan Manger

**Affiliations:** UC San Diego Radiation Medicine and Applied Sciences, 3855 Health Sciences Dr. #0843, La Jolla, CA 92093-0843 USA

**Keywords:** SBRT, Lung cancer, Radiotherapy, Gradient index, Gradient measure, Retrospective

## Abstract

**Background:**

The treatment of lung lesions with stereotactic body radiation therapy calls for highly conformal dose, which is evaluated by a number of metrics. Lung stereotactic body radiation therapy clinical trials constrain a plans gradient index. The purpose of this work is to describe the dependence of clinically achievable dose gradient on planning target volume.

**Methods:**

Three hundred seventy-four lung stereotactic body radiation therapy treatment plans were retrospectively reviewed and selected for this study. The relationship between R50% and planning target volume size was observed and compared against the RTOG 0915 and 0813 constraints noting minor and major deviations. Then a least squares regression was used to determine the coefficients for a power functional form of the dependence of gradient measure (GM) on planning target volume size.

**Results:**

Of the 317 peripheral lung SBRT plans, 142 exhibited no deviation, 135 exhibited a minor deviation, and 40 exhibited a major deviation according to the RTOG 0915 dosimetric.

conformality and dose fall-off constraints. A plot of gradient measure versus planning target volume size for peripheral lesions, excluding RTOG 0915 major deviations, is fit with an power function of GM = 0.564 V^0.215^.

**Conclusions:**

Using the PTV size and GM relationship we have characterized, treatment plans with PTV < 85 cm^3^ can be evaluated subjectively to our previously plans, and given a percentile GM. This relationship and evaluation is useful for volumetric modulated arc therapy lung stereotactic body radiation therapy treatment planning and quality control.

**Electronic supplementary material:**

The online version of this article (10.1186/s13014-019-1334-9) contains supplementary material, which is available to authorized users.

## Background

In radiation oncology, stereotactic body radiation therapy (SBRT) for lung lesions is an external beam radiation therapy technique that utilizes precise targeting and dose delivery of radiation with acceptable toxicity [1]. The ablative target doses delivered with SBRT are modeled after intracranial stereotactic radiosurgery (SRS). Unlike conventionally fractionated radiation therapy, which achieves the therapeutic window through the relative radiosensitivity of tumor tissue compared to normal tissue, the stereotactic approach achieves the therapeutic window with geometric accuracy and a highly conformal dose distribution. [2–4]

Lung SBRT is particularly challenging due to physiological organ and target motion (respiration). The necessary geometric accuracy has been achieved by utilizing advances in patient immobilization, tumor motion assessment, and near real time imaging studies at the time of treatment [5–7]. The high dose per fraction makes steep dose gradients desirable. A good plan quality is characterized with highly conformal dose distribution and steep dose gradients nearly isotropically around the target. Previously, volumetric modulated arc therapy (VMAT) has been shown to offer improved target conformality with shorter treatment times for lung SBRT with both coplanar and non-coplanar delivery over conventional 3D conformal treatments [8–10]. An optimal lung SBRT plan achieves target dose conformality while avoiding excessive high dose and intermediate dose spillage. For example, the conformality of a plan is characterized with the conformity index (CI), which is a ratio of the prescription isodose volume, PIV, i.e. the volume encompassed by the 100% isodose line (IDL), and the volume of the planning target volume (PTV) [11]. Lung SBRT clinical trials aim for a CI less than 1.2 and utilize a number of other dose metrics [12, 13]. Gradient index (GI) is a tool to evaluate intermediate dose fall off, and is the ratio of the volume of half the prescription isodose and the PIV [14]. The clinically achievable GI is dependent on the size of the PTV [13]. R50% is a similar quantity presented in the RTOG 0813 and 0915 lung SBRT protocols [12, 13], and it is defined as the ratio of the volume of the 50% isodose volume and the PTV volume.

The Eclipse (Varian, Palo Alto, CA) treatment planning system reports gradient measure (GM), which is defined as the difference, in centimeters, of the equivalent sphere radii of the 50 and 100% prescription IDL volumes [15]. Similar to the GI and R50%, this metric has value in assessing the high dose fall off; but unlike GI or R50%, the dependence of clinically achievable GM on PTV size for lung SBRT has not yet been established. The aim of this work is to characterize the clinically achievable GM dependence on PTV size across multiple radiation oncology clinics for the purpose of dosimetric quality control.

## Methods

Clinically approved treatment plans were retrospectively reviewed and selected for this study. All plans utilized a coplanar volumetric modulated arc therapy (VMAT) technique with one isocenter per target receiving SBRT treatment in one to five fractions. Treatments were collected across four centers within our institution and planned in accordance with the guidelines of RTOG 0813 or 0915 depending on its location – central or peripheral (> 2 cm from the proximal bronchial tree). The treatments were planned with Varian Eclipse versions 11 and 13.6, using the Analytical Anisotropic Algorithm (AAA) (versions 11 or 13.6) for dose calculation with a grid size of 0.25 cm. All plans were treated on either a Varian TrueBeam or C-Series linear accelerator with a Millennium 120 multileaf collimator (MLC). All plans used 6 MV energy, but some used the higher dose rate 6X-SRS mode, and one of the machines used the flattening filter free 6X energy (6X-FFF).

The Varian Eclipse Scripting application programming interface (API) was used to extract treatment and planning quality metrics from each selected case. Specifically, treatment date, center, disease site and location (central and peripheral), prescribed dose, number of fractions, number of fields, monitor units (MU), PTV size (cm^3^), effective diameter, gradient measure, gradient index, R50%, conformity index, mean dose, max dose, minimum PTV dose, and percent of the PTV receiving 100% of the prescription dose (V100) were extracted or calculated. We then analyzed the relationships between these parameters.

The relationship between R50% and PTV size was observed and compared against the RTOG 0915 and 0813 constraints noting minor and major deviations. Next, the relationship between GM and PTV size was investigated. Least squares regression was used to determine the coefficients for a linear, exponential, logarithmic, and power functional form of the dependence of GM on PTV size. Linear and exponential functional forms had low R^2^ values (0.762 and 0.696), while a logarithmic functional form had a better R^2^ value (0.823), but residuals that did not appear to be randomly distributed. To achieve the greatest R^2^ value (0.842) and random residuals distribution, a power functional form was selected and is presented in Eq. 1, where GM is the gradient measure in cm, V is the PTV volume in cm^3^, and A and B are the unknown coefficients.

1 $$ \mathrm{GM}=\mathrm{A}{\mathrm{V}}^{\mathrm{B}} $$

Quantiles regression, which is a more robust method than least squares regression when there are outliers in the data, was also used to determine coefficients for Eq. 1 for the 10, 25, 50, 75, and 90% quantiles. All regression analyses were performed using R version 3.5.1 [16].

## Results

**Fig. 1:**
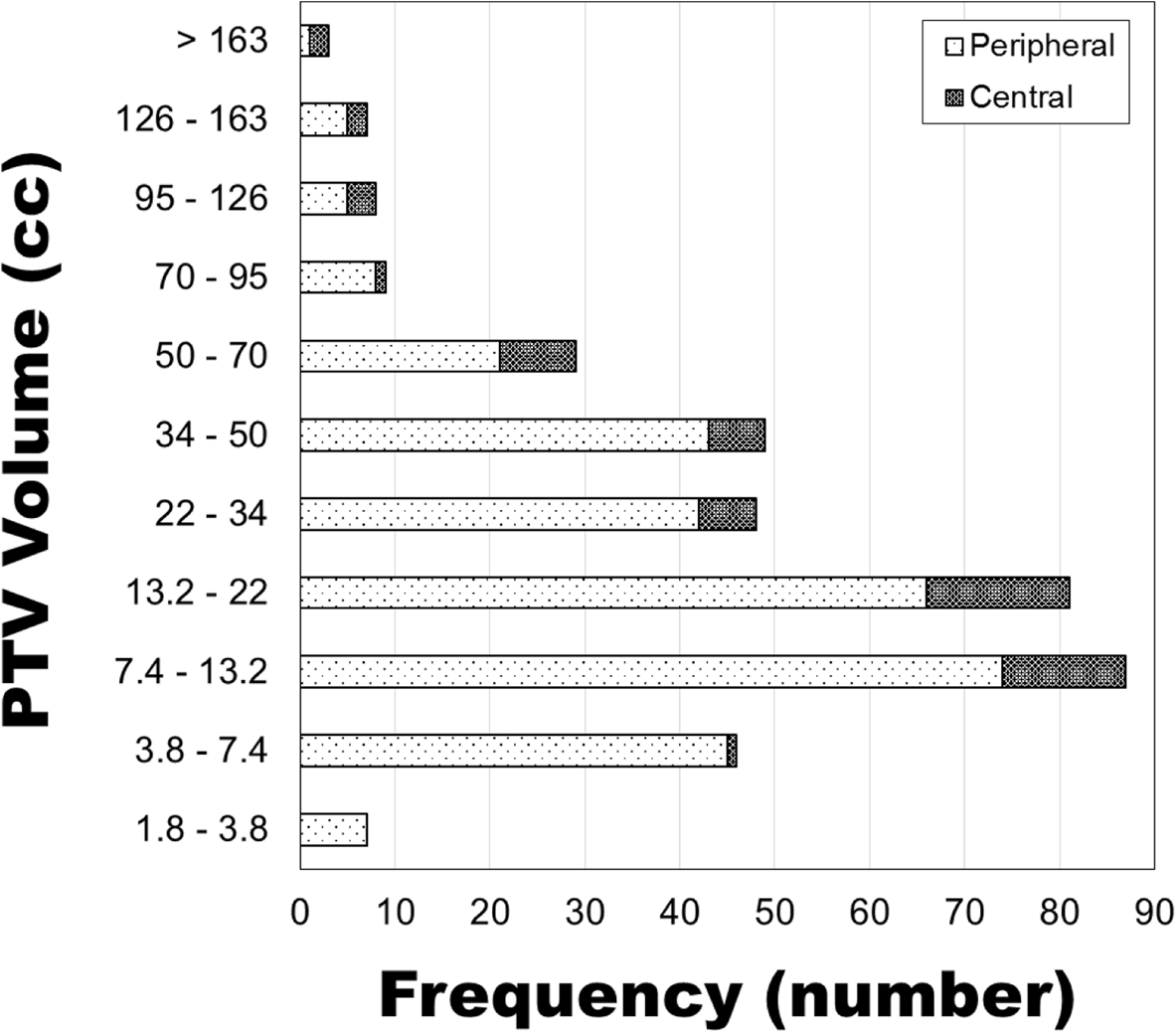
Distribution of the size of all PTVs in this study. PTV volume is presented using the RTOG 0915 volume bins. Data are separated between centrally located (within 2 cm of airways or mediastinal pleura) and peripheral

From January 2016 through March 2018, 374 lung SBRT plans were identified – 317 peripheral (85%) and 57 central (15%). Central was defined as being within a 2 cm radius of the airway or mediastinal pleura. PTV volumes ranged from 2.05 to 310.45 cc. A frequency distribution of the PTV volumes is shown in Fig. 1. Averages of the data binned using PTV volume bins from RTOG 0915 are presented in Table 1.

**Table 1:** Statistics for all lung SBRT plans included in this study. n is the number of plans in the volume bin. Conformity index is the quotient of the volume receiving the prescribed dose and the PTV volume. Prescription (Rx) Dose is the prescribed dose normalized such that V_100_ ≥ 95%. IMRT factor is the quotient of total monitor units and fractional dose in cGy

Volume bin (cm^3^)	n	PTV Volume (cm^3^)	Gradient Measure (cm)	R50%	Conformity Index	Rx Dose (cGy)	Fractions	IMRT factor
1.8–3.8	7	3.05	0.84	8.59	1.09	3871	3.6	3.7
3.8–7.4	46	5.72	0.86	5.99	1.09	4626	3.5	3.2
7.4–13.2	87	9.93	0.94	5.14	1.05	4726	4.0	3.1
13.2–22	81	17.55	1.06	4.73	1.03	4808	4.4	3.0
22–34	48	26.53	1.14	4.30	1.01	4884	4.3	2.8
34–50	49	41.11	1.28	4.07	1.00	4814	4.5	2.9
50–70	29	58.35	1.33	3.75	1.00	4855	4.7	2.8
70–95	9	81.93	1.45	3.62	0.99	4250	4.4	3.2
95–126	8	108.08	1.65	3.72	0.98	4562	5.0	2.6
126–163	7	143.05	1.88	4.07	0.99	4686	4.9	2.5
> 163	3	235.67	2.12	3.61	1.01	4267	4.7	3.0

**Fig. 2:**
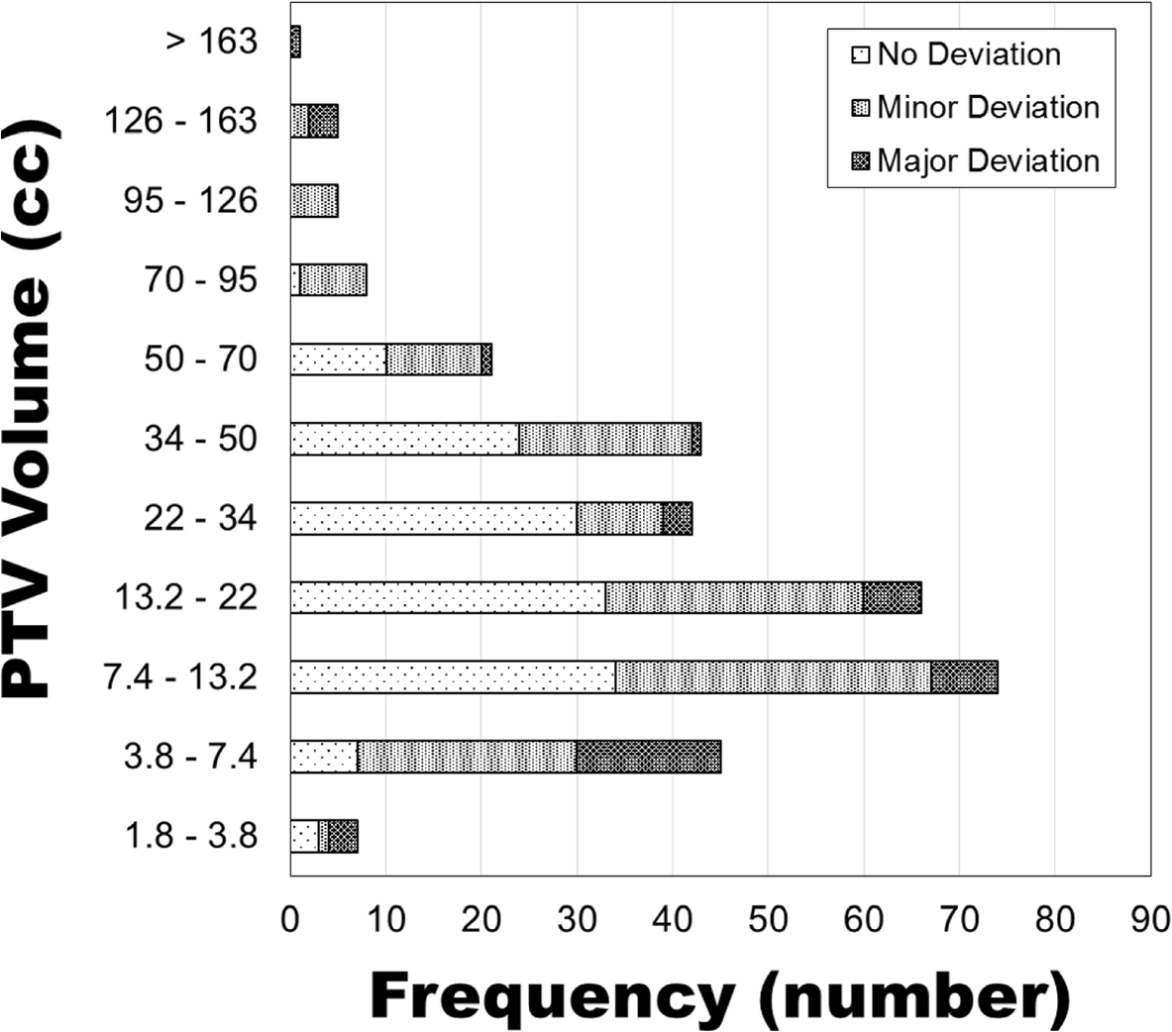
Plan performance of all PTVs relative to RTOG 0915 dosimetric conformality and dose falloff constraints for peripheral lesions

Of the 317 peripheral lung SBRT plans, 142 exhibited no deviation, 135 exhibited a minor deviation, and 40 exhibited a major deviation according to the RTOG 0915 dosimetric conformality and dose falloff constraints. Plan performance relative to RTOG 0915 dosimetric conformality and dose falloff constraints is presented in Fig. 2 for each PTV volume bin.

**Fig. 3:**
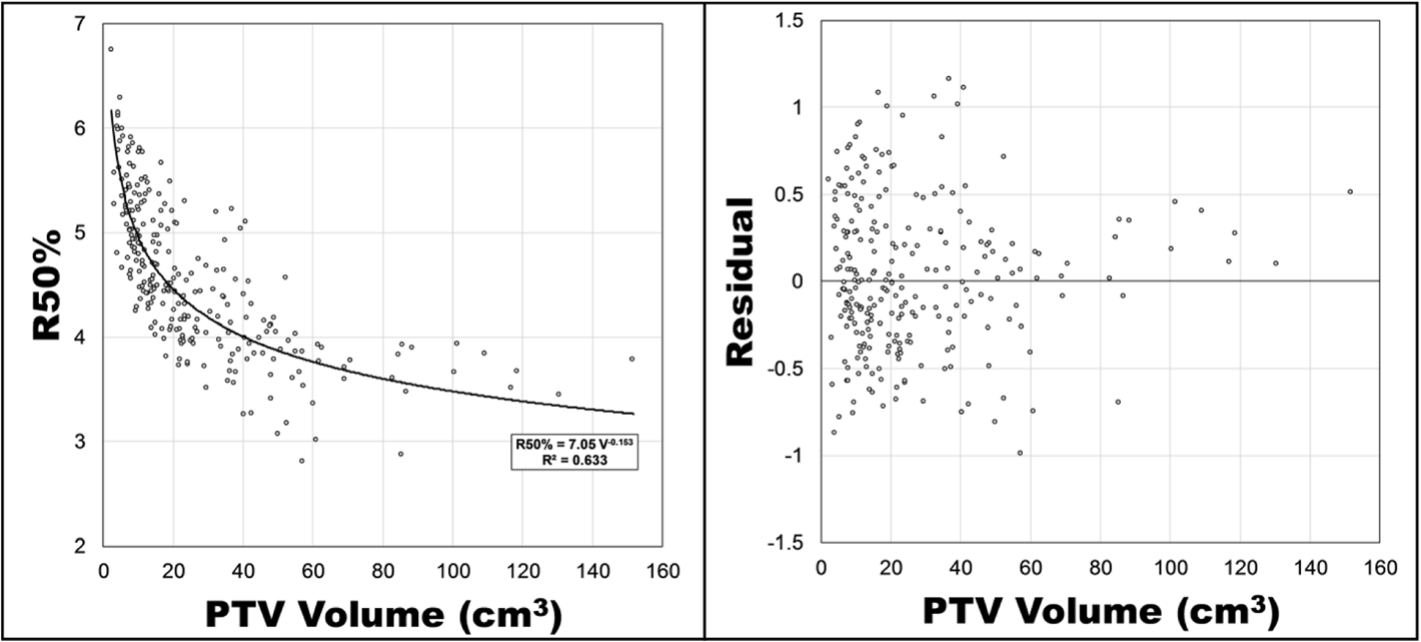
R50% versus the PTV volume for peripheral lesions, excluding RTOG 0915 major deviations. A least squares fit of a power function is presented along with its functional form and R [2]. Residuals of the predicted R50% minus the actual R50% are presented on the right

There were 277 protocol-acceptable peripheral lung SBRT plans. A plot of R50% versus PTV volume (cm^3^) is presented in Fig. 3. Included with that figure is a power function fit using least squares regression and a plot of its residuals. The functional form of the relationship between PTV volume, V (cm^3^) and R50% for peripheral lesions is

2 $$ \mathrm{R}50\%=7.05{\mathrm{V}}^{-0.153} $$

with a standard error of 0.021 and 0.007 for the A and B parameters and a coefficient of determination, R [2], of 0.63. The residuals plot appears random up to a PTV volume of approximately 85 cm^3^ (Additional files 1 and 2). Above 85 cm^3^, Eq. 2 consistently predicted a smaller R50% than what was calculated in the clinically approved, protocol-acceptable plans.

**Fig. 4:**
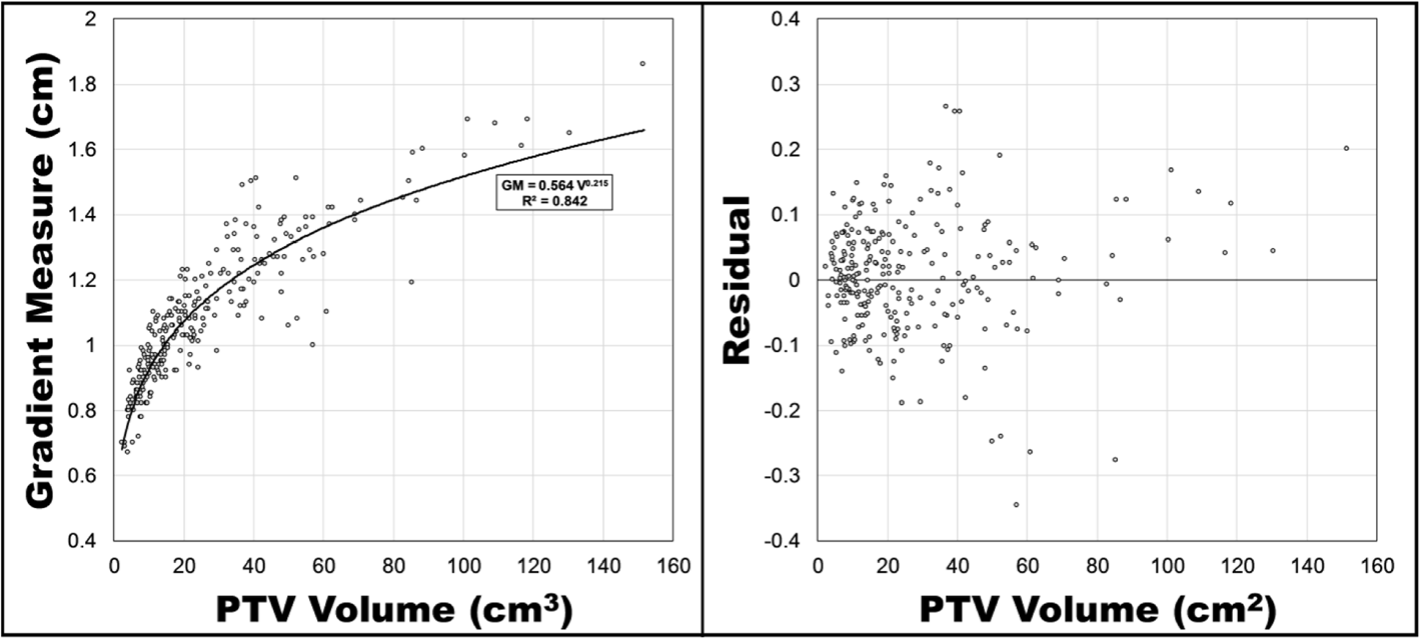
Gradient measure versus PTV volume for peripheral lesions, excluding RTOG 0915 major deviations. A least squares fit of a power function is presented along with its functional form and R [2]. Residuals of the predicted gradient measure minus the actual gradient measure are presented on the right

A plot of gradient measure versus PTV volume for peripheral lesions is presented in Fig. 4 with a power function fit using least squares regression. The functional form of that relationship is

3 $$ \mathrm{GM}=0.564{\mathrm{V}}^{0.215} $$

with a standard error of 0.017 and 0.006 for the A and B parameters and an R^2^ value of 0.850. A plot of the residuals is also included in Fig. 3, and as was the case with R50%, Eq. 3 predicted a smaller gradient measure than what was achieved clinically. The improved coefficient of determination in Eq. 3 signifies that it can explain a greater percent of the random variation of Gradient Measure than Eq. 2 can explain of R50%. A notable limitation of Eqs. 2 and 3 is their predictability for PTV volumes of approximately 85 cm^3^ and greater. Additional plots of the gradient measure versus PTV volume for all peripheral lesion plans including major deviations (n = 317) and all central lesion plans including major deviations (n = 57) are included in additional files.

**Fig. 5:**
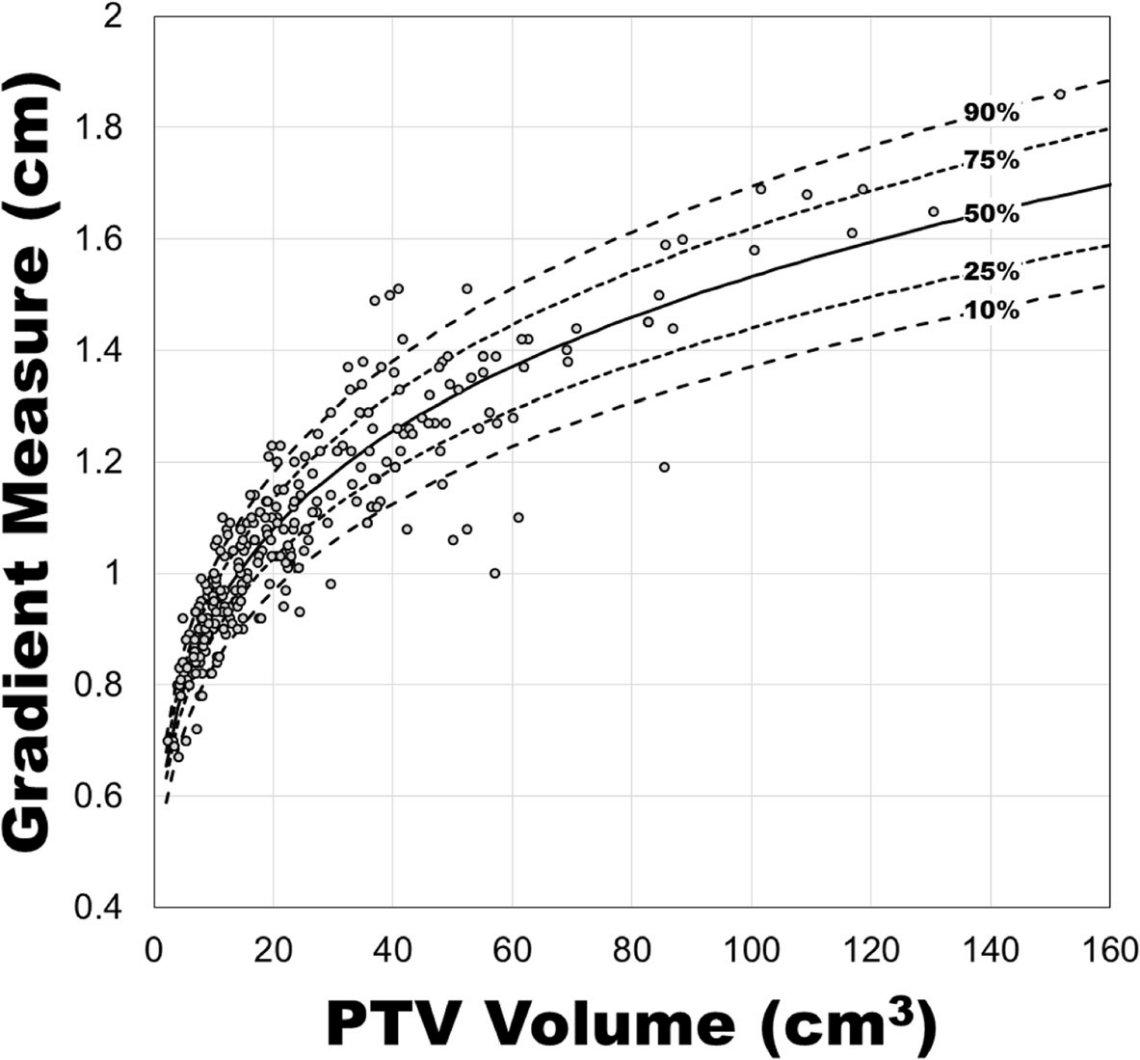
Gradient measure versus PTV volume along with quantiles regression curves for the 90th, 75th, 50th, 25th, and 10th quantiles, for peripheral lesions, excluding RTOG 0915 major deviations

Quantiles regression [17] was performed for the 90th, 75th, 50th, 25th, and 10th percentiles on the relationship between GM and PTV volume for peripheral lesions (Fig. 5). In this case, 90th percentile means that 90% of the plans had a Gradient Measure equal to or lower than that value, so the lower the percentile the steeper the high dose falloff. The coefficients for each of the percentile curves is presented in Table 2.

**Table 2:** Coefficients for the 10th, 25th, 50th, 75th, and 90th percentile curves with the functional form of Eq. 1 for peripheral lesions, excluding RTOG 0915 major deviations. The standard error for each coefficient is included in parentheses

Percentile (%)	A	B
90	0.507 (0.059)	0.216 (0.022)
75	0.547 (0.026)	0.210 (0.009)
50	0.562 (0.017)	0.218 (0.006)
25	0.583 (0.021)	0.222 (0.007)
10	0.602 (0.020)	0.225 (0.007)

## Discussion

A predictable relationship exists between PTV volume and gradient measure or R50% for protocol-acceptable, peripheral lung SBRT plans at our institution. Its functional form is presented in Eqs. 2 and 3. A limitation of these equations is their tendency to under-predict the gradient measure and R50% for large PTV volumes (⪆ 85 cm^3^). Eight of the 277 peripheral lung SBRT plans had a PTV volume greater than 85 cm^3^. A separate function could be fit to the larger PTV data, but more treatment plans are required in this volume range.

Narayanasamy et al. [18] have studied the relationship between R50% and PTV volume for a sample size of 105 lung SBRT plans. In their paper, the relationship between R50% and PTV volume was found to be

4 $$ \mathrm{R}50\%=7.2{\mathrm{V}}^{-0.13} $$

with an R^2^ of 0.58. This formula predicts a similar, but larger R50% (less steep dose dropoff) than the one presented in this work (Eq. 2), which is likely due to the plans being from another institution with different planning policies and procedures. Their planning techniques were a mix of 3DCRT, sliding window IMRT, and RapidArc, while this work only considered RapidArc plans.

The functional form of the GM and PTV size relationship offers lung SBRT treatment planners a tool to evaluate the GM of their plan beyond a simple “no deviation, minor deviation, or major deviation” described in clinical trials. Additionally, the quantiles regression allow planners to estimate the percentile of a plan’s GM, so they may develop an understanding of the greatest plan outliers and the potential GM increase from replanning a treatment.

**Fig. 6:**
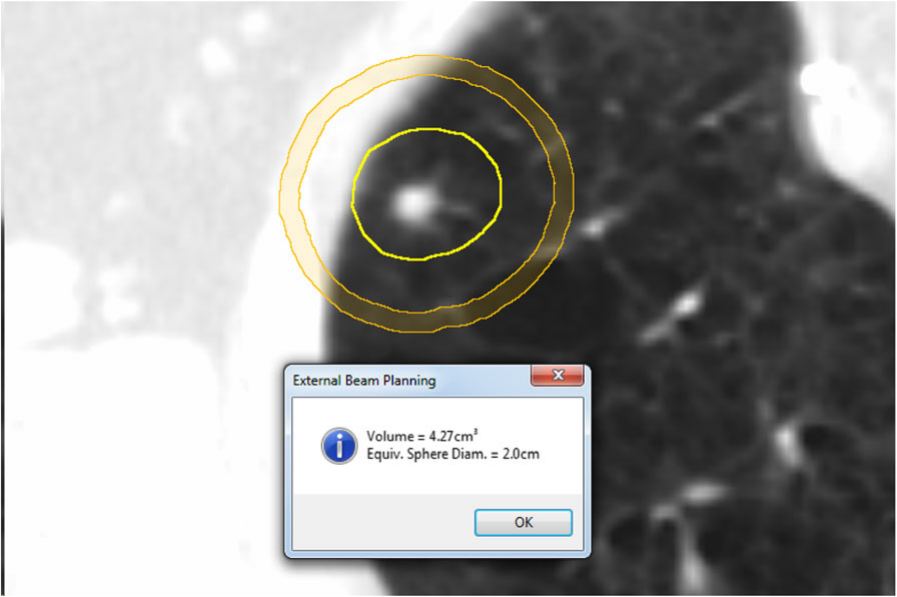
An example of how to use PTV volume to guide optimization. In this case, the PTV volume is measured, and the predicted gradient measure is determined for that volume. A ring structure is created with an inner radius that is 1 GM from the PTV and an outer radius that is 3 mm larger (3 mm thick rind). This ring is used to control the 50% IDL

Additionally, the results presented in this work can be used prospectively during treatment planning to inform the creation of a planning psudo-structure to reduce GM. Since the CI is near unity for most RapidArc plans at our institution, the average distance from the edge of the PTV to the 50% isodose line is approximately the Gradient Measure. This relationship can be used to create control structures for the purpose of minimizing R50%. Future work will explore a proposed workflow would be as follows: 1. Planner calculates the 25th or 10th percentile gradient measure given the PTV volume; 2. Planner creates a bespoke control ring (Fig. 6) with an inner dimension 1 GM from the PTV (the thickness of this ring is set such that the ring is continuous – 3 mm for a high resolution structure in Eclipse); 3. For optimization purposes, the control ring has an upper constraint of 0% receiving 50% of the prescription dose with a priority equal to the lower constraint for PTV coverage; 4. After calculation, the planner and physicist benchmark the plan against the gradient measure from Eq. 3. As part of plan QC, the percentile curves presented in Table 2 may be used to determine how the plan performed relative to the plans in this dataset. Since the planner is aiming for a lower gradient measure and R50%, the lower the percentile the steeper the dose falloff. Naturally, the plan should be compared against any institutional normal tissue constraints and the RTOG 0915 dosimetric conformality and dose falloff constraints.

As shown in the residuals plot, there is variability that is likely from a source other than PTV volume, such as PTV shape, risk structure location, and user variability. More advanced algorithms such as knowledge based planning (KBP) [19] may prove to have a better predictability than the rudimentary method proposed in the previous paragraph since it considers the geometric relationship between the target and nearby organs at risk. However, advanced algorithms such as KBP are not yet widely available or implemented into routine use in the clinic, so this simpler approach should prove helpful for treatment planning and quality control in those settings.

## Conclusion

PTV size can be used to predict the gradient measure for PTVs less than approximately 85 cm^3^. This relationship is useful for RapidArc peripheral lung SBRT treatment planning and quality control purposes when more advanced algorithms such as KBP aren’t available.

## Additional files

Additional file 1: **Figure S1.** Gradient measure versus PTV volume for all peripheral lesion plans (*n* = 317) including major deviations. A least squares fit of a power function is presented along with its functional form and R [2]. (TIF 1714 kb)

Additional file 2: **Figure S2**. Gradient measure versus PTV volume for all central lesion plans (*n* = 57) including major deviations. A least squares fit of a power function is presented along with its functional form and R [2]. (TIF 1586 kb)

## Data Availability

The datasets used and/or analyzed during the current study are available from the corresponding author on reasonable request.

## Abbreviations

AAA: Analytical anisotropic algorithm
API: Application programming interface
CI: Conformity index
GI: Gradient index
GM: Gradient measure
IDL: Isodose line
KBP: Knowledge based planning
MLC: Multileaf collimator
MU: Monitor unit
PTV: Planning target volume
SBRT: Stereotactic body radiation therapy
SRS: Stereotactic radiosurgery
VMAT: Volumetric modulated arc therapy

## References

[1] Benedict SH (2010). Stereotactic body radiation therapy: the report of AAPM task group 101. Med Phys.

[2] Chang BK, Timmerman RD (2007). Stereotactic body radiation therapy: a comprehensive review. Am J Clin Oncol.

[3] Benedict SH, Lin PS, Zwicker RD, Huang DT, Schmidt-Ullrich RK (1997). The biological effectiveness of intermittent irradiation as a function of overall treatment time: development of correction factors for linac-based stereotactic radiotherapy. Int J Radiat Oncol Biol Phys.

[4] Kavanagh BD (2003). How should we describe the radioblologic effect of extracranial stereotactic radlosurgery: equivalent uniform dose or tumor control probability?. Med Phys.

[5] Korreman S S (2015). Image-guided radiotherapy and motion management in lung cancer. The British Journal of Radiology.

[6] Principles and Practice of Image-Guided Radiation Therapy of Lung Cancer. *CRC Press* (2017). Available at: https://www.crcpress.com/Principles-and-Practice-of-Image-Guided-Radiation-Therapy-of-Lung-Cancer/Cai-Chang-Yin/p/book/9781498736732. (Accessed 6th Aug 2018).

[7] Caillet V, Booth JT, Keall P (2017). IGRT and motion management during lung SBRT delivery. Phys Med.

[8] Timmerman RD, Forster KM, Chinsoo Cho L (2005). Extracranial stereotactic radiation delivery. Semin Radiat Oncol.

[9] Matuszak MM, Yan D, Grills I, Martinez A (2010). Clinical Applications of Volumetric Modulated Arc Therapy. Int J Radiation Oncol Biol Physics.

[10] Verbakel WFAR, Senan S, Cuijpers JP, Slotman BJ, Lagerwaard FJ (2009). Rapid delivery of stereotactic radiotherapy for peripheral lung tumors using volumetric intensity-modulated arcs. Radiother Oncol.

[11] Landberg T, et al. ICRU Report 62: prescribing, recording and reporting photon beam therapy (Supplement to ICRU Report 50). J ICRU. 1999;32(1):13.

[12] Bezjak A (2012). Seamless phase I/II study of stereotactic lung radiotherapy (SBRT) for early stage, centrally located, non-small cell lung cancer (NSCLC) in medically inoperable patients. RTOG.

[13] Videtic G (2012). A randomized phase II study comparing 2 stereotactic body radiation therapy (SBRT) schedules for medically inoperable patients with stage I peripheral non-small cell lung cancer. RTOG.

[14] Paddick I, Lippitz B (2006). A simple dose gradient measurement tool to complement the conformity index. J Neurosurg.

[15] Eclipse Photon and Electron 15.5 Reference Guide. Varian Med Syst. 2017;1–575.

[16] R Core Team. R: a language and environment for statistical computing. Vienna: R Foundation for statistical computing; 2013. URL http://www.R-project.org/. Accessed 10 Oct 2018.

[17] Koenker R, Hallock KF (2001). Quantile regression. J Econ Perspect.

[18] Narayanasamy G (2018). Technical note: a planning technique to lower normal tissue toxicity in lung SBRT plans based on two likely dependent RTOG metrics. Med Phys.

[19] Good D, Lo J, Lee WR, Wu QJ, Yin FF, Das SK (2013). A knowledge-based approach to improving and homogenizing intensity modulated radiation therapy planning quality among treatment centers: an example application to prostate cancer planning. Int J Radiat Oncol Biol Phys.

