# Supplementary figures and images for: Lung Stereotactic Body Radiation Therapy (SBRT) dose gradient and PTV volume: a retrospective multi-center analysis
Source: Radiat Oncol. 2019 Sep 3;14:162. doi: 10.1186/s13014-019-1334-9 (PMC6724320; doi:10.1186/s13014-019-1334-9)

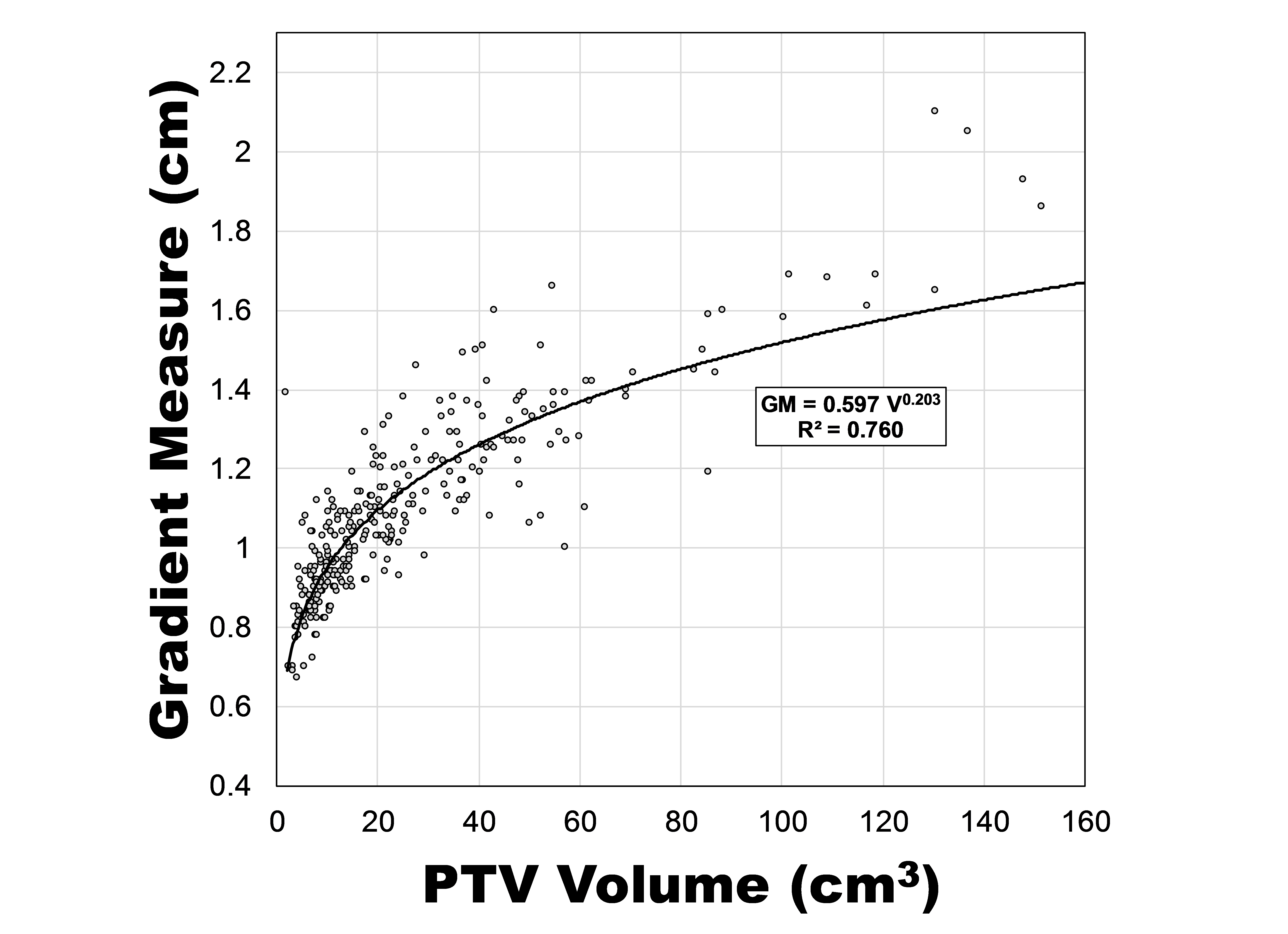

Supplement: Supplementary file 1 — Figure S1. Gradient measure versus PTV volume for all peripheral lesion plans (n = 317) including major deviations. A least squares fit of a power function is presented along with its functional form and R [2]. (TIF 1714 kb) [file 13014_2019_1334_MOESM1_ESM.tif]

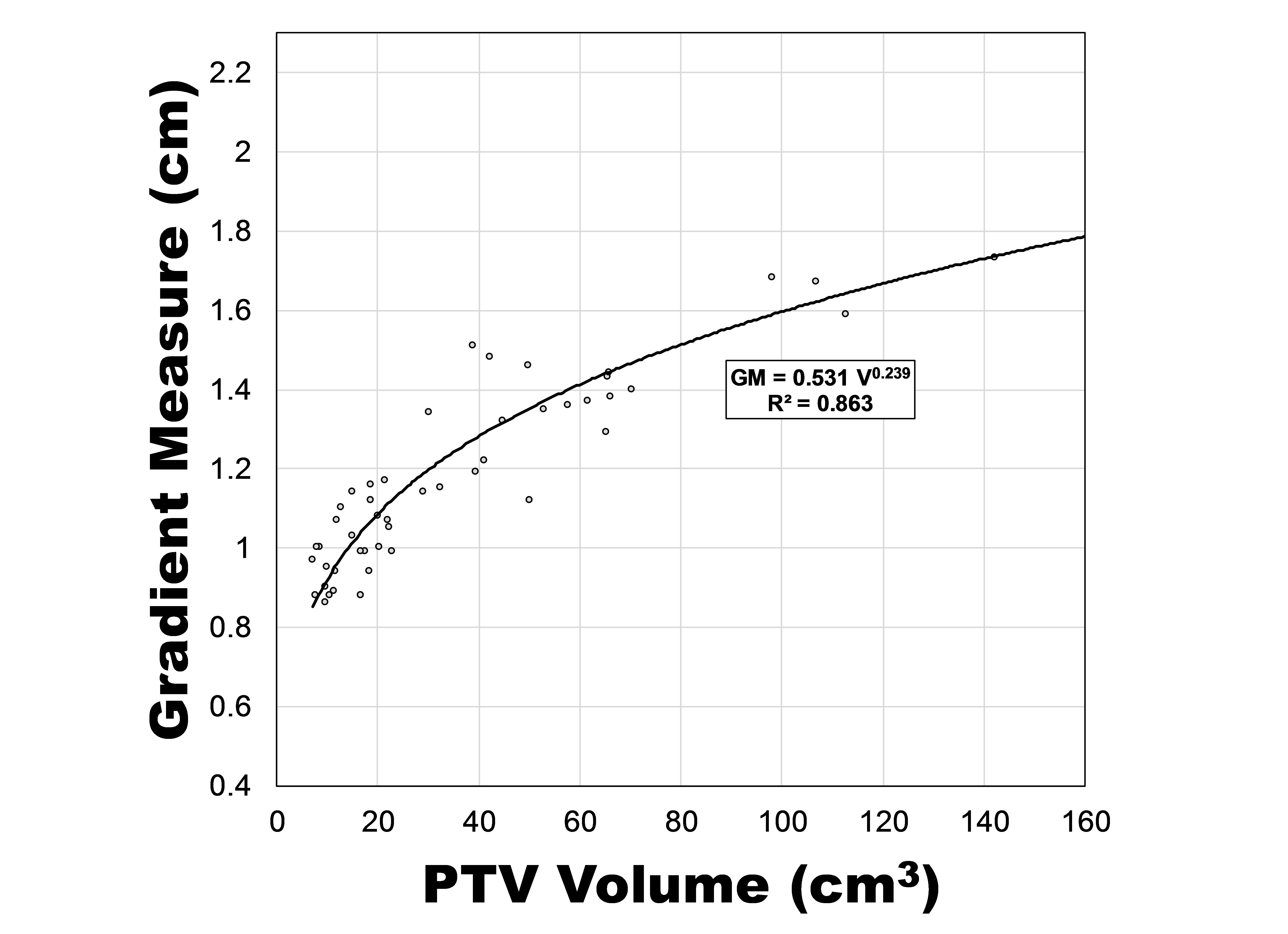

Supplement: Supplementary file 2 — Figure S2. Gradient measure versus PTV volume for all central lesion plans (n = 57) including major deviations. A least squares fit of a power function is presented along with its functional form and R [2]. (TIF 1586 kb) [file 13014_2019_1334_MOESM2_ESM.tif]
